# Construction and characterization of metal ion-containing DNA nanowires for synthetic biology and nanotechnology

**DOI:** 10.1038/s41598-019-43316-1

**Published:** 2019-05-06

**Authors:** Simon Vecchioni, Mark C. Capece, Emily Toomey, Le Nguyen, Austin Ray, Alissa Greenberg, Kosuke Fujishima, Jesica Urbina, Ivan G. Paulino-Lima, Vitor Pinheiro, Joseph Shih, Gary Wessel, Shalom J. Wind, Lynn Rothschild

**Affiliations:** 1 Department of Biomedical Engineering, Columbia University, New York, NY 10027 USA; 2 Department of Chemistry, Stanford University, Stanford, CA 94305 USA; 3 Department of Structural Biology, Stanford University School of Medicine, Stanford, CA 94305 USA; 4 Department of Electrical Engineering and Computer Science, Massachusetts Institute of Technology, Cambridge, MA 02139 USA; 5 School of Engineering, Brown University, Providence, RI 02912 USA; 6 Department of Chemical Engineering, Stanford University, Stanford, CA 94305 USA; 7 Department of History, Stanford University, Stanford, CA 94305 USA; 8 Earth-Life Science Institute, Tokyo Institute of Technology, Meguro-ku, Tokyo 152-8550 Japan; 9 Geology, Minerals, Energy, & Geophysics Science Center, U.S. Geological Survey, Menlo Park, CA 94025 USA; 10 Planetary Science Branch, NASA Ames Research Center, Moffett Field, CA 94035 USA; 11 Blue Marble Space Institute of Science, NASA Ames Research Center, Planetary Systems Branch, Moffett Field, CA 94035-0001 USA; 12 Institute of Structural and Molecular Biology, University College London, London, WC1E 6BT UK; 13 Department of Natural Sciences and Mathematics, University of Saint Mary, Leavenworth, KS 66048 USA; 14 Department of Molecular Biology, Cell Biology and Biochemistry, Brown University, Providence, RI 02912 USA; 15 Department of Applied Physics and Applied Mathematics, Columbia University, New York, NY 10027 USA

**Keywords:** Synthetic biology, Bionanoelectronics

## Abstract

DNA is an attractive candidate for integration into nanoelectronics as a biological nanowire due to its linear geometry, definable base sequence, easy, inexpensive and non-toxic replication and self-assembling properties. Recently we discovered that by intercalating Ag^+^ in polycytosine-mismatch oligonucleotides, the resulting C-Ag^+^-C duplexes are able to conduct charge efficiently. To map the functionality and biostability of this system, we built and characterized internally-functionalized DNA nanowires through non-canonical, Ag^+^-mediated base pairing in duplexes containing cytosine-cytosine mismatches. We assessed the thermal and chemical stability of ion-coordinated duplexes in aqueous solutions and conclude that the C-Ag^+^-C bond forms DNA duplexes with replicable geometry, predictable thermodynamics, and tunable length. We demonstrated continuous ion chain formation in oligonucleotides of 11–50 nucleotides (nt), and enzyme ligation of mixed strands up to six times that length. This construction is feasible without detectable silver nanocluster contaminants. Functional gene parts for the synthesis of DNA- and RNA-based, C-Ag^+^-C duplexes in a cell-free system have been constructed in an *Escherichia coli* expression plasmid and added to the open-source BioBrick Registry, paving the way to realizing the promise of inexpensive industrial production. With appropriate design constraints, this conductive variant of DNA demonstrates promise for use in synthetic biological constructs as a dynamic nucleic acid component and contributes molecular electronic functionality to DNA that is not already found in nature. We propose a viable route to fabricating stable DNA nanowires in cell-free and synthetic biological systems for the production of self-assembling nanoelectronic architectures.

## Introduction

Nanoelectronics has become a transformative technology for both industrial and personal computing applications, yet the limitations of traditional manufacturing methods make current prototypes expensive and difficult to mass produce^1^. Nanowires produced by common lithographic techniques requiring pattern transfer and vapor deposition suffer from high resource cost, population heterogeneity, lack of structural precision, and difficulty of construction^2–8^. Consequently, conventional nanowire synthesis often requires prohibitively complex and expensive manufacturing infrastructure. In light of these obstacles, the use of DNA as an alternative nanowire would be pivotal; with its linear configuration, alterable binding sites and sequence-defined geometry, the structure of DNA can be reliably controlled and replicated at low relative cost, and can be dynamically manipulated in multiple ways, including through chemical linkers^9–11^, ligand-aptamer operations^12–14^, fluorescent markers^15,16^, and nanoparticle/nanotube attachment^17–19^. Furthermore, through the addition of short staple sequences or branching components, DNA can be used as a template for molecular origami to self-assemble a variety of precise nanostructures^20–23^. While other naturally conducting biological nanowires and hybrids have been developed, including bacterial pili, coated nanoparticles, metalated protein filaments etc.^24–29^, DNA remains at least an order of magnitude smaller at a diameter of 2 nm, a size scale appropriate for future semiconductor device integration at sub-10 nm production nodes^30,31^. Relative to inorganic nanowire production, the annealing temperatures of DNA are significantly lower, the reagents to construct them far less toxic, and the technical requirements greatly reduced. As in any biosynthetic system, there is the potential for rapid, cheap, and environmentally-safe mass production from cellular components that can be easily tuned for different purposes. Furthermore, this system does not require the heavy infrastructure utilized for inorganic production, and thus it has the potential for distributed manufacturing in low-resource environments, including in space, the moon or Mars.

Despite these advantages, native DNA has yet to demonstrate robust functionality as a molecular wire. Measurements of DNA conductivity have shown behavior ranging from insulating^32,33^ to induced-superconducting^34^. This disparity in behavior is likely due to technical difficulties in performing electrical measurements on individual molecules^32,35–41^. Still, efforts to enhance the conductivity of DNA are ongoing through various metalization schemes, including M-DNA (metal DNA) formation through the non-specific exchange of imino protons for metal ions^42^; gold^43,44^, palladium^45^, and cobalt^46^ nanocluster attachment using azide-alkyne interactions or reduction-based schemes; nanoparticle-catalyzed formation of E-DNA (eccentric DNA) in GC-dominated duplexes^47^; nanosphere assembly from polycytosine oligonucleotides assuming an i-motif (cytosine quadruplex) configuration^48^; and site-specific thiol functionalization in rolling circle amplification^49^, and DNA origami^17,50,51^.

**Figure 1:**
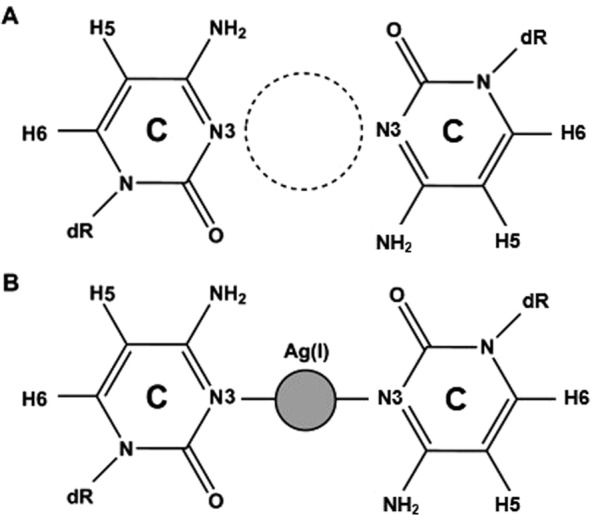
Structure of the C-C mismatch predicted from NMR studies^57^: (**A**) unbound with gap; (**B**) bound to Ag^+^ at N3 position to form the dC:Ag^+^:dC metal base pair.

Metal-nucleobase interactions have been studied since the 1960s when an affinity was discovered between the mercury cation (Hg^2+^) and thymine-enriched DNA polynucleotides^52^, but several decades passed before the notion of introducing a metal ion directly into a DNA duplex was first described in detail by Tanaka and coworkers in 2002^53^. This was followed by work that demonstrated the replacement of Watson-Crick G-C base pairs with mismatched cytosine pairs capable of forming a coordinating bond with Ag^+^ ^54,55^. Due to the smaller size of pyrimidine bases relative to their purine cognates, the mispairing of two cytosines introduces a 4.4 Å gap in the helical structure which selectively incorporates Ag^+^ (Fig. 1) in a reaction energetically driven by the dehydration of the cation^56,57^. Consequently, the introduction of cytosine-mismatch repeats into oligonucleotide sequences allows functionalization of the helical core with a chain of single metal ions that is surrounded by π-stacking nucleobase rings and stabilized by the sugar-phosphate backbone. Recent studies suggest that in ion-coordinating pyrimidine systems, a metallophilic bond between axially-adjacent cations may cause a compression in the inter-pair bond length of B-form DNA from 3.4 Å to 3.3 Å^58,59^. This potential attraction in the Ag^+^ system suggests an interaction between the valences of the two ions and an open pathway for electron mobility. Long coordinating ion chains in both linear duplexes and ring-like species have been developed with great promise by several groups for their attractiveness as a nanowire^60–63^.

In line with this work, we recently demonstrated a significant increase in single molecule conductivity with a scanning probe (STM) break junction experiment in Ag^+^-functionalized duplexes^41^. The conductivity of a Ag^+^-intercalated polycytosine mismatch duplex C_11_-(Ag^+^)_11_-C_11_ was compared to that of a Watson-Crick duplex G_11_-C_11_, resulting in a 10-fold higher conductivity for the Ag^+^-intercalated polycytosine mismatch duplex. Watson-Crick DNA helices conduct electrons through delocalized π-orbitals. Since guanine has the lowest ionization potential of any nucleobase^64^, higher conductivity than a polyguanine sequence indicates that C_11_-(Ag^+^)_11_-C_11_ uses an alternate conduction scheme, likely through the ion chain itself. This finding makes Ag^+^-intercalated, polycytosine mismatch duplexes promising as potential biological nanowires, motivating the need for complete characterization of their physical and chemical properties. Here we describe structural, thermodynamic, and kinetic experiments that confirm the stability of Ag^+^-intercalated polycytosine mismatch duplexes. We demonstrate that these functionalized duplexes can be constructed with a great degree of tunability, and we successfully polymerize these constructs using a variety of methods to achieve long DNA nanowires. Given their great potential in both biological and nanoscale devices, we propose a viable route to fabricating stable DNA nanowires for nanoelectronic applications in cell-free and synthetic biological systems.

## Methods

### DNA sample preparation

All unmodified and fluorescently-labeled DNA oligonucleotides were synthesized by Elim Biopharmaceuticals (Hayward, CA, USA) and Integrated DNA Technologies (Coralville, IA, USA), respectively (Table 1). Strand alignment and ion intercalation sites are shown diagrammatically in Figs S1 and S2. Lyophilized oligonucleotides were suspended at 100 μM in deionized water as a working solution. Oligonucleotides were annealed at final concentrations of 2 μM in 10 mM MOPS (C_7_H_15_NO_4_S) [pH 7.2] and 100 mM NaNO_3_ with various concentrations of AgNO_3_ specified in the text at 95 °C, followed by cooling to 4 °C over 2 hr^65^.

**Table 1:** Oligonucleotide sequences used in this study.

**C11**. 11 nt polycytosine	
Template	5′-CCCCCCCCCCC-3′
**C20**. 20 nt polycytosine	
Template	5′-CCCCCCCCCCCCCCCCCCCC-3′
**C30**. 30 nt polycytosine	
Template	5′-CCCCCCCCCCCCCCCCCCCCCCCCCCCC-3′
**T30**. 30 nt polythymine	
Template	5′-TTTTTTTTTTTTTTTTTTTTTTTTTTTTTT-3′
**Oligo A**. 32bp10CC duplex	
Template	5′-TTATATTTACCACCTCCTCCACCTTTTAGATT-3′
Complement	5′- AATCTAAAACCTCCACCACCTCCTAAATATAA-3′
**Oligo B**. 50bp6CC duplex	
Template	5′-TAAACCACTCATACCACAACAACTCTCTACTCCTACACATCATCCATCTC-3′
Complement	5′-GAGATGGATCATGTCTAGGACTAGAGAGTTCTTGTGGTATCAGTCGTTTA-3′
**Oligo C**. 25bp1CC duplex palindrome	
Template	5′-TATTAAATAAAACTTTTATTTAATA-3′
**Oligo D**. 26bp0CC canonical duplex	
Template	5′-CATTAATGCTATGCAGAAAATCTTAG-3′
Complement	5′- CTAAGATTTTCTGCATAGCATTAATG-3′
**Hairpin A**. 19bp8CC hairpin	
Template	5′-biotin-TTTGTGT(Cy3)CTCCTTCATCCCTCACTTTGTATGCAAACTCACCCATCAACCAGACA-Cy5-3′

### Nuclear magnetic resonance

Gradient-selected ^1^H-^1^H correlation spectroscopy (COSY) experiments were performed on a 600 MHz Varian UNITY INOVA spectrometer (Agilent Technologies, Santa Clara, CA, USA) with a 5 mm ^1^H{^13^C, ^15^N} Z-PFG conventional probe at 25 °C. These homonuclear COSY spectra report on the presence and electronic environment of proton nuclei that are connected to one another by two (^2^J_HH_ coupling) or three (^3^J_HH_ coupling) covalent bonds. For DNA molecules, protons that meet these criteria are cytosine base protons H5 and H6 and all deoxyribose protons. Intercalation of silver cations between the N3 atoms of mismatched cytosines perturbs the electronic environment of both cytosines’ H5 and H6 protons, resulting in an observable change to the COSY spectra. In this study, all COSY spectra were acquired with 32 scans, 1024 t_2_ points, and 128 t_1_ points. FIDs were Fourier transformed using squared-sinebell weighting and post-processed with MestReNova (Mestrelab Research, Santiago de Compostela, Spain).

NMR samples were prepared by suspending the model AT-rich, single-cytosine-mismatch, palindromic, DNA 25-mer sequence Oligo C in 10 mM K_3_PO_4_, 75 mM NaNO_3_, and 99.99% D_2_O to a concentration of 3.84 mM. COSY spectra were acquired of the original duplex, after metalation by addition of AgNO_3_ to 8 mM, and after de-metalation by precipitation of Ag^+^ with excess NaCl and dialysis against the original buffer without AgNO_3_. Dialysis was performed using Slide-A-Lyzer MINI dialysis cups with 3.5 kDa molecular weight cutoff (Thermo Fisher Scientific, Waltham, MA, USA) through three buffer exchanges in 24 h intervals with stirring at 6 °C. Before each NMR experiment, the sample was reannealed by melting at 95 °C for 15 min and cooling to 6 °C over a period of 2 h.

### FRET

To measure Förster resonance energy transfer (FRET) between Cy3 and Cy5 dual-labeled DNA, we employed a real-time zero-mode-waveguide (ZMW)-based DNA sequencer (RS) platform (Pacific Biosciences, Menlo Park, CA, USA) that was modified for use in non-sequencing fluorescent systems^66^. The RS is capable of illuminating the 150,000 ZMWs on the sample chip (Pacific Biosciences) with green (532 nm) and red (642 nm) lasers while monitoring fluorescent emission on timescales ranging from milliseconds (single frame) to several hours. This instrument has been used to study a variety of biological pathways, ranging from DNA-DNA duplex formation^66^ to extricating individual dynamic steps involved in translation^67^.

The DNA sequence used in the FRET experiments was the 52-base Hairpin A (see Table 1). The T7–T26 and A33–A52 regions were intentionally designed to be devoid of G in order to promote the formation of up to nine C-C mismatches flanked by canonical A-T pairs, and a central gTATGc tetraloop was present to encourage intrastrand helix formation. Sample chips were prepared by coating with neutravidin and immobilizing 25 μM of the biotinylated DNA in assay buffer (10 mM Tris [pH 8.25], 2.5 mM 3,4-dihydroxybenzoic acid, 250 nM protocatechuate dioxygenase^68^, and 2.5 mM TSY triplet-state quencher solution [Pacific Biosciences, Menlo Park, CA, USA]). At this concentration of DNA, approximately 13% of the wells were occupied during the experiment, thereby promoting single-molecule occupancy within the wells exhibiting fluorescence. During the experiments, either 1 mM NaNO_3_ or AgNO_3_ in the assay buffer was mechanically delivered to the sample chip while the sample was illuminated by only the green (532 nm) laser at a power of 0.48 μW/μm^2^. Emission measurements for both Cy3 and Cy5 fluorophores were recorded for each waveguide at 100 ms intervals for a total observation period of 10 min. The detection of Cy5 emission indicated that energy was transferred from excited Cy3 to ground state Cy5 through the process of FRET.

After acquisition, raw data was automatically filtered for co-localization of both green (Cy3) and red (Cy5) emission above five standard deviations of noise in Matlab (MathWorks, Natick, MA, USA). Manual curation of the filtered dataset allowed a representative set of 200 time-resolved emission curves (traces) to be extracted at random for each delivery experiment; each trace represented one DNA molecule and indicated FRET through simultaneous loss of Cy3 emission intensity and gain of Cy5 emission intensity. To calculate the FRET efficiency in each trace, the Cy3 emission intensity during FRET was internally normalized to the maximum intensity without FRET and minimum intensity after photobleaching. Traces were then categorized into one of eight evenly-distributed FRET efficiency value ranges, and Gaussian fits of the resultant histogram data were performed. To calculate the Ag^+^-dependent DNA folding rate, the data set was purged of traces in which FRET was observed prior to AgNO_3_ delivery, and the molecules exhibiting FRET at a given frame were categorized into 5-s intervals. Equation 1 describes the exponential fit of the population of molecules exhibiting high FRET (peak at 0.75) as a function of time. From this equation, it was possible to deduce the rate of Ag^+^-dependent DNA folding (parameter b) in the high-FRET state when f(t) = 0.75. Using this value of b, the rate of DNA folding unrelated to Ag^+^ incorporation (fit parameter k) was calculated by solving a double-exponential fit (Eq. 2) to the total population of molecules in which FRET was detected (function g) as a function of time.

1 $${\rm{f}}({\rm{t}})={\rm{a}}(1-{{\rm{e}}}^{-{\rm{bt}}})+{\rm{c}}$$

2 $$g(t)=d(1-{e}^{-bt})+h(1-{e}^{-kt})+lt$$

### Melting temperature analysis

An annealed volume of 20 μL of 2 μM double-stranded DNA oligonucleotides was diluted into 1 mL MOPS buffer in a crystal cuvette on a Perkin-Elmer Lambda 950 UV spectrophotometer (PerkinElmer, Inc., Shelton, CT, USA) with Peltier temperature control. Absorbance relative to the MOPS buffer blank was measured at 280 nm. Measurements were carried out using a D_2_ lamp with 2-nm spectral bandwidth and 1-cm path length. Readings were collected across a temperature range of 25–100 °C with 60-s pauses at each temperature point to achieve thermal equilibrium. Five measurements in 1-s intervals were recorded at each point and averaged. Melt curves were constructed in GraphPad Prism (Origin Labs, Northampton, MA, USA), and the melting temperatures were extracted from a generalized Hill equation, or five-parameter logistic (5PL) regressions. Equation 3 describes the normalized absorbance (Y) as a function of temperature (independent variable T), which was fit using the absorbance extremes (Y_min_ and Y_max_), the Hill slope (fit parameter H), the temperature at half-maximum absorbance (melting temperature T_m_), and an asymmetry coefficient (fit parameter S) to account for clipping of the curves near 100 °C. Equation 4 is provided to simplify the exponent in the final expression for Y(T). Control samples without injection of Ag^+^ were normalized, in identical buffer and concentration, to the absorbance start point (fit parameter Y_min_) of the Ag^+^ experimental condition, such that the relative change was correlated to that of the experimental sample. This analysis yields a relative change of 0.1% in the ion-free control, with an F-value of non-zero fit of 0.0108.

3 $$Y(T)=({Y}_{max}-{Y}_{min})/{(1+{10}^{H(Z-T)})}^{S}$$

4 $$Z={T}_{m}+\frac{1}{H}\,\mathrm{log}({2}^{1/S}-1)$$

### Spectral analysis for nanoclusters

UV absorption spectra were collected over a range of 250–700 nm on a Lambda 950 UV spectrophotometer (PerkinElmer, Inc.) at room temperature. Measurements were carried out using a D_2_ lamp with spectral bandwidth of 0.05–5.00 nm and a path length of 1 cm. Sample absorbance relative to the empty buffer was collected in wavelength intervals of 1 nm across the spectrum. DNA sequences annealed with and without Ag^+^ were compared in cytosine-enriched sequences (C11, C20, C30, Hairpin A (Table 1)) and in canonically-paired sequences (Oligo D, CG20 (Table S1)).

To strip Ag^+^ ions from 2 μM DNA samples after annealing, NaCl was added to a concentration of 50 mM and was allowed to react at room temperature for 15 min before pelleting the AgCl precipitate with a tabletop centrifuge (12,000 g, 12 min). To force reduction of solvent accessible Ag^+^ ions, NaBH_4_ was added to 2 μM duplexes to achieve a final reductant concentration of 1 mM. Samples were then shaken vigorously for 2 min at 6 °C and left to react at room temperature overnight. Spectra were collected both before and after precipitation of AgCl and reduction by BH_4_^−^.

### Phosphorylation and enzymatic ligation

For 3′ phosphorylation, 5 μL each of 2 μM template and complement strands for Oligo D were annealed with a saturating amount of Ag^+^ and mixed with 3 μL of MilliQ-purified water, 1 μL (10 U) of T4 phosphonucleokinase (PNK) (Life Technologies, Carlsbad, CA, USA, Cat. #EK0031), and 1 μL (1 unit) of 10X T4 DNA Ligase Buffer (New England Biolabs, Ipswitch, MA, USA) and incubated for 60 min at 37 °C. End-ligation was achieved by subsequently adding 1 μL of T4 DNA Ligase (New England Biolabs) and incubating for 2 hr at 25 °C. Nondenaturing PAGE gels were prepared via standard techniques^69^. Staining was performed either using SYBR Gold (Life Technologies), silver stain kit (Thermo Fisher), toluidine blue (Thermo Fisher), or strand functionalization with FAM fluorophores (Elim Biopharamceuticals), and gels were visualized on a Typhoon Trio gel scanner (Amersham Biosciences, Amersham, UK).

### Biobrick design

The functional region of gene part BBa_K1218026, including the oligonucleotide sequences of interest, the BioBrick prefix and suffix, and the expression primers, was synthesized by Elim Biopharmaceuticals and cloned into plasmid backbone pSB1C3 (BioBricks Foundation, San Francisco, CA, USA) using an EcoRI-PstI (New England Biolabs, #E0546S) cloning protocol for BioBrick assembly^70^. Part BBa_K1218022 was synthesized by GeneBlocks (Integrated DNA Technologies) and cloned into pSB1C3 using the same protocol. Part BBa_K1218022 served as the template for PCR amplification of DNA and RNA nanowires Hairpin B1 and B2 (Table S1). This BioBrick was utilized through PCR amplification of the functional region using forward and reverse primers 5′-CAACCATACGACACGCCTC-3′ and 5′-ACCTCACCGACTCAGCC-3′, respectively. The DNA nanowire product was subsequently isolated by PmeI excision (R0560S, New England Biolabs), annealed in the manner described above in the presence of 24-fold excess AgNO_3_ with respect to oligonucleotide molarity, and purified by PAGE. The template for RNA Hairpin B2 (Table. S1) was instead excised by digestion with EcoRV (R0195S, New England Biolabs), and the RNA nanowire itself was synthesized in a cell-free system by transcription with T7 RNA polymerase (E2040S, New England Biolabs). The RNA nanowire was annealed in the presence of 24-fold excess AgNO_3_ and purified by PAGE. BioBrick BBa_K1218026 was designed to allow PCR amplification using forward and reverse primers 5′-CCAAGCACGCCCACCT-3′ and 5′-TGGTAGGTGGCGGTGC-3′, respectively, in order to generate DNA nanowire 32bp10CC Oligo A. This product was annealed with a 100:1 molar ratio between AgNO_3_ and oligo in the manner described above. Additional documentation may be found on the BioBrick registry^71,72^.

## Results

### Characterization of Ag^+^ intercalation into DNA duplexes

**Figure 2:**
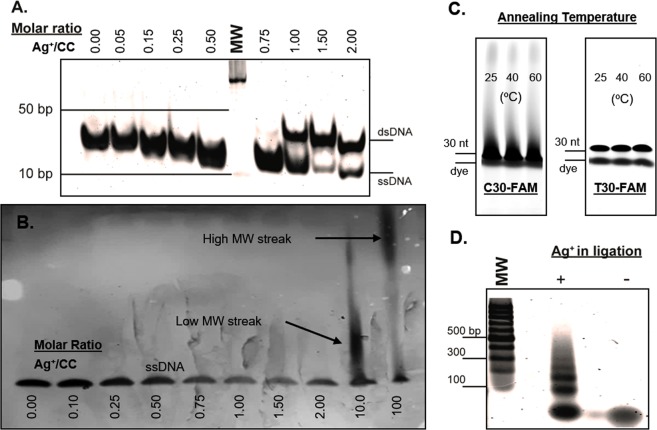
Polyacrylamide gels showing formation of nanowires of tunable lengths. (**A**) Equimolar dependence of Ag^+^ and mismatches is shown in end-clamped, fixed-annealing-frame, mismatched Oligo A. (**B**) Polycytosines are generally ill-behaved; here ion excess promotes formation of sticky end regions and long-chain polymerization into high-MW regions for C30. (**C**) Ten-fold ion excess produces multimeric polymerization in FAM-labeled C30 at low temperatures of 25 °C, 40 °C and 60 °C, while FAM-labeled T30 controls show no such effect. (**D**) Successful phosphorylation and end-ligation of 50-bp Oligo B is exhibited of up to 300 bp. Uncropped gels for this figure can be found in Fig. S3.

Thermal annealing of cytosine-mismatched DNA duplexes from cytosine-enriched DNA oligomers subjected to varying salt conditions was readily visualized by mass distribution using polyacrylamide gel electrophoresis (PAGE) (Fig. 2). Using two complementary 32 bp ssDNA sequences with ten cytosine mismatch points (Oligo A) as a model, we found that strand annealing is directly proportional to the concentration of Ag^+^ ions in the reaction (Fig. 2A). The sequence of Oligo A was designed with a (CCN)_x_ pattern (where N is any canonically-pairing DNA nucleotide) to produce a duplex with a repeating motif of two C-Ag^+^-C pairs followed by one Watson-Crick standard pair; thus, the duplex is tailored to accommodate exactly 10 Ag^+^ ions (1 Ag^+^ ion per CC mismatch (Figs S1 and S2)). Nine Watson-Crick terminal pairs on each end served as a clamp to force alignment of the central mismatch motif. Oligo A duplexes were first observed at a Ag^+^:CC molar ratio of 0.75, and a solid duplex with a fixed molecular weight is seen at molar unity (Fig. 2A). Apparent band shift from 0.05 to 0.75 Ag^+^ occupancy is likely a gel artifact and is also observed in the tilt of the 10 bp ladder band at that position. Similar band shifts from the left to the right can be seen in other uncropped polyacrylamide gels run in the same gel box (Fig. S2A,C).

**Figure 3:**
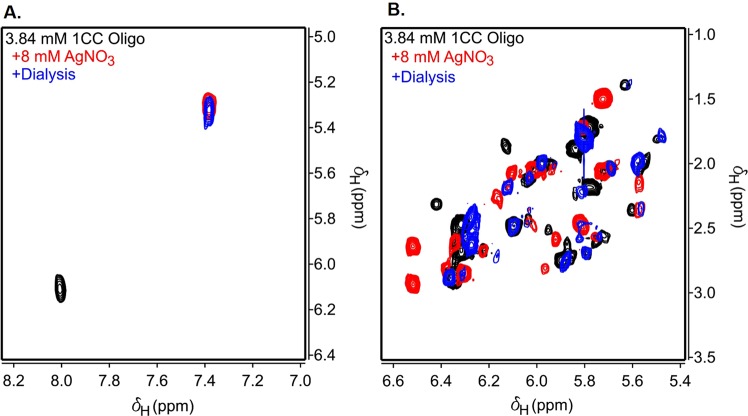
(**A**) COSY NMR spectrum of Oligo C, highlighting the ^3^J_HH_-coupled cytosine H5-H6 crosspeak. In phosphate buffer, the cytosine H5 and H6 chemical shifts are 6.11 and 8.01 ppm, respectively (black contours). The addition of AgNO_3_ produces an upfield translation of the chemical shifts to 5.30 and 7.38 ppm, respectively (red contours). This shift is preserved after unbound Ag^+^ ions were precipitated with 50 mM NaCl, and the DNA was dialyzed by equilibration with phosphate buffer (blue contours). (**B**) COSY spectrum of crosspeaks corresponding to backbone ^3^J_HH_-coupled deoxyribose H1′-H2′ and H1′-H2″ crosspeaks. AgNO_3_ greatly perturbs the shifts in this region, but many of the backbone peaks revert to their pre-AgNO_3_ shifts after dialysis.

When a similar analysis of molar ratio annealing was performed on the C30 oligonucleotide, a 30-nt polycytosine DNA sequence, a markedly different result is achieved. Unlike Oligo A, which featured 9 bp Watson-Crick “clamps” at both termini, C30 lacks a defined pairing frame and may anneal in a range of thermodynamically-promiscuous configurations (Figs S1 and S2). As a result, instead of producing a unitary duplex, C30 strands interlock into chains whereby overhanging nucleotides from one duplex can pair with the overhangs from another as a function of Ag^+^ ion concentration (Fig. 2B). In these experiments, annealing is not observed until AgNO_3_ is added to a 10:1 Ag^+^:CC ratio, producing an ill-defined, multi-band streak representing a distribution of chained C30 strands. An even higher molecular weight streak is apparent at a 100:1 Ag^+^:CC ratio, suggesting that C30 chain interlocking is driven by Ag^+^ ion availability. This effect was also reproduced in longer polycytosines (Fig. S4). Multimeric polycytosine streaking can be achieved preferentially with excess environmental Ag^+^, even at temperatures far below 100 °C, if given sufficient reaction time (Fig. 2C). Though the single-stranded bands are more prominent than those produced by high-temperature conditions in Fig. 2B, streaking into arbitrarily high molecular weight regions of the gel can be observed even when annealed at room temperature for times over 48 h. A solution NMR structure of a Ag^+^-intercalated, single-cytosine mismatch DNA duplex was recently described^57^. Using a similar AT-rich model DNA duplex, we employed ^1^H-^1^H COSY to visualize Ag^+^ intercalation by cytosine H5-H6 chemical shifts^56^. In DNA, cytosine H5 and H6 peaks are usually found within the chemical shift ranges of 5.60 ± 0.60 ppm and 7.50 ± 0.50 ppm, respectively, and these nuclei are the only protons within the four canonical DNA bases that exhibit ^3^J_HH_ coupling. To confirm that Ag^+^ ions are being incorporated between the strands of a palindromic 25 bp DNA duplex with a single cytosine mismatch (Oligo C), we acquired COSY spectra of the duplex before and after the addition of AgNO_3_ (Fig. 3A). In the absence of Ag^+^ ions, the duplex produces the anticipated single cytosine H5-H6 crosspeak corresponding to H5 and H6 chemical shifts of 6.11 ppm and 8.01 ppm, respectively. Although two cytosines exist within the duplex, the palindromic nature of the sequence means that both cytosines are exposed to identical chemical shift environments, thereby producing a single peak (Fig. S1). Spiking AgNO_3_ to excess caused a significant perturbation in the chemical shifts of the H5 and H6 protons, moving their chemical shifts upfield to 5.30 ppm and 7.38 ppm, respectively. Since only a single crosspeak is produced, the duplex is not disrupted by the incorporation of the Ag^+^ ion.

In addition to the cytosine H5-H6 crosspeak, within the spectral window of Fig. 3B, ^3^J_HH_ couplings between deoxyribose H1′ (5.5 ± 0.5 ppm) and H2′/H2″ (2.5 ± 1.0 ppm) are observed, although the chemical shift dispersion is too poor for definitive assignments. However, these signals can be aggregated as reporters of deoxyribose backbone deformations due to Ag^+^ ion incorporation. From this window, we recorded 15–20 H1′-H2′/H2″ crosspeaks in the control without added AgNO_3_. In the presence of AgNO_3_, however, approximately twelve of these H1′-H2′/H2″ crosspeaks had clear chemical shift perturbations caused by the Ag^+^ ions. These spectra suggest that Ag^+^ ions not only intercalate within the duplex but also associate with the negatively-charged phosphate backbone.

**Figure 4:**
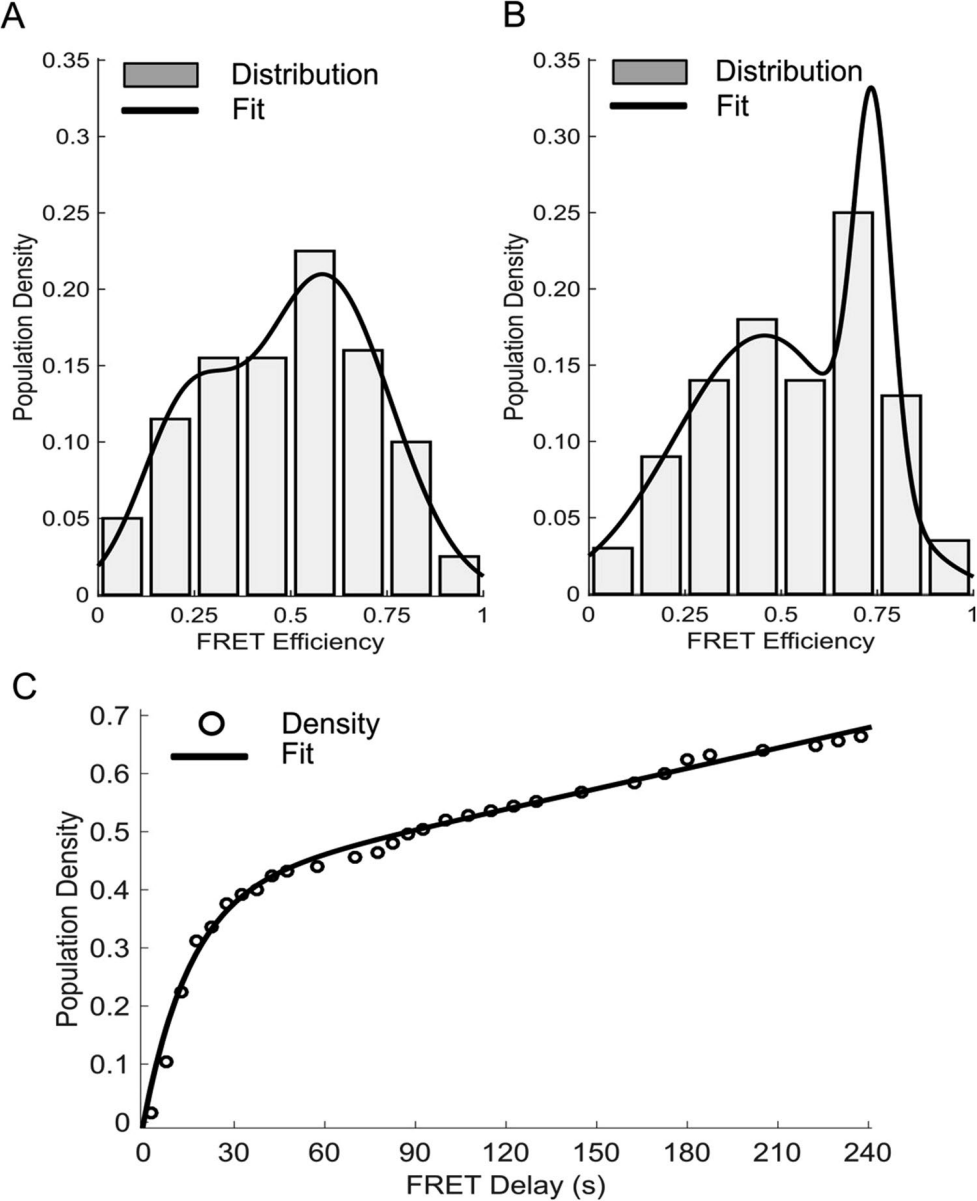
(**A**) Histogram of FRET intensity values for the NaNO_3_ control of Hairpin A fit to a sum of two Gaussian functions centered at 0.227 and 0.587 intensities. (**B**) Histogram of FRET intensities for the AgNO_3_ delivery to Hairpin A with a double-Gaussian fit of peaks 0.457 and 0.738. (**C**) Extraction of the FRET delay parameter (time until FRET) for only the AgNO3-delivered high FRET traces. The folding rate for the Ag-dependent structure is approximated from the exponential fit to be 0.065 s^−1^.

We then investigated the kinetics of Ag^+^ incorporation into polycytosine DNA sequences by real-time single molecule fluorescence. A 52-nt DNA sequence was synthesized with Cy3 and Cy5 fluorophores near the 3′ and 5′ termini, respectively, and a central gTATGc tetraloop (Hairpin A (Fig. S1)). The sequence was designed such that Ag^+^-induced cytosine mismatch pairing would allow the otherwise-unstructured DNA strand to adopt a hairpin conformation, thereby enabling FRET energy transfer from the Cy3 donor to the Cy5 acceptor molecule, with an efficiency inversely correlated with the sixth power of the inter-fluorophore distance. In the absence of AgNO_3_, Hairpin A molecules adopted conformations with FRET values whose populations can be represented as two overlapping Gaussian distributions of low (0.227 ± 0.119) and medium (0.587 ± 0.173) FRET efficiencies, respectively (Fig. 4A). The broadness of the distributions indicates substantial conformational heterogeneity indicative of weak base pairing. Of these molecules in which FRET was observed, the vast majority folded before the delivery of NaNO_3_ and did not change conformations after delivery. When AgNO_3_ was delivered to Hairpin A, we recorded FRET values in a very different population distribution: one low-FRET Gaussian at 0.457 ± 0.234 and one high-FRET Gaussian at 0.738 ± 0.049 (Fig. 4B). The high-FRET population is significantly larger than that of the low-FRET and has a narrow range of FRET values. In contrast to the medium FRET conformation adopted in the absence of AgNO_3_, the high-FRET state is recorded exclusively after AgNO_3_ delivery and appears slowly over the period of observation, signifying that Ag^+^ ions are, indeed, participating in DNA folding. None of the traces in which the high FRET state was recorded were found to directly interconvert with the Ag^+^-independent medium FRET state, so base pairing in the medium FRET state must be broken prior to refolding into the high FRET state. Furthermore, it is likely that the high-variance Gaussian centered at 0.457 in the AgNO_3_-delivered sample is simply the composite of both Ag-independent low and medium FRET states, which fold prior to AgNO_3_ delivery and have sufficiently long lifetimes that the Ag-induced high FRET state is only produced from the remaining single-stranded DNA molecules.

To calculate the nucleotide folding rate, the observation frame in which FRET was first recorded for the DNA molecules exposed to AgNO_3_ was extracted, and the molecules that had folded into low-FRET conformations prior to AgNO_3_ delivery were removed. The rate of Ag^+^-induced folding is approximated as the exponential accumulation of molecules exhibiting FRET from the remaining population of molecules (Fig. 4C). From this fit, we have estimated the Ag^+^-induced folding rate to be 6.5 ± 0.7 × 10^−2^ s^−1^. Inputting this rate manually and resolving for the Ag^+^-independent folding rate among the entire population of molecules reveals the rate of folding into flexible, Ag^+^-absent conformations to be approximately 15 s^−1^, which is typical for conformational rearrangement of DNA molecules^73^. Since Ag^+^-dependent Hairpin A folding is 230 times slower than that of the Ag^+^-independent conformations, the energy barrier for the incorporation of 9 Ag^+^ ions must be approximately 13.3 ± 0.5 kJ/mol. Molecular dynamics simulations have calculated that ion detainment onto charged biomolecules has an activation energy of 2–5 kJ mol^−1^ ion^−1^ ^74^, so an energy barrier on the order of 10^1^ kJ/mol is a plausible estimate for Ag^+^ ion incorporation into DNA base pairs.

### Stability of Ag^+^-bound DNA duplexes

The viability of Ag^+^-intercalated DNA duplexes as biological nanowires necessitates thermodynamic and structural stability in a range of environmental conditions. To this end, we subject a variety of duplexes to thermal and chemical stress in order to assess the behavior of mismatched Ag^+^-intercalated nanowires. First, we attempted to decompose the metalated AT-rich, single-mismatch Oligo A DNA duplex by precipitating Ag^+^ ions with excess NaCl, followed by cold dialysis into the original phosphate buffer. COSY spectra revealed that this process did not revert the cytosine H5-H6 crosspeak to its unmetalated chemical shifts (Fig. 3A). However, the backbone deoxyribose shifts did largely revert back to the Ag^+^-absent control spectrum when Ag^+^ ions were removed by precipitation and dialysis (Fig. 3B). Since only the deoxyribose chemical shifts were restored to unmetalated values upon Ag^+^ removal, Ag^+^ ions intercalated between cytosine mismatches must be highly stable and protected from the solvent.

**Figure 5:**
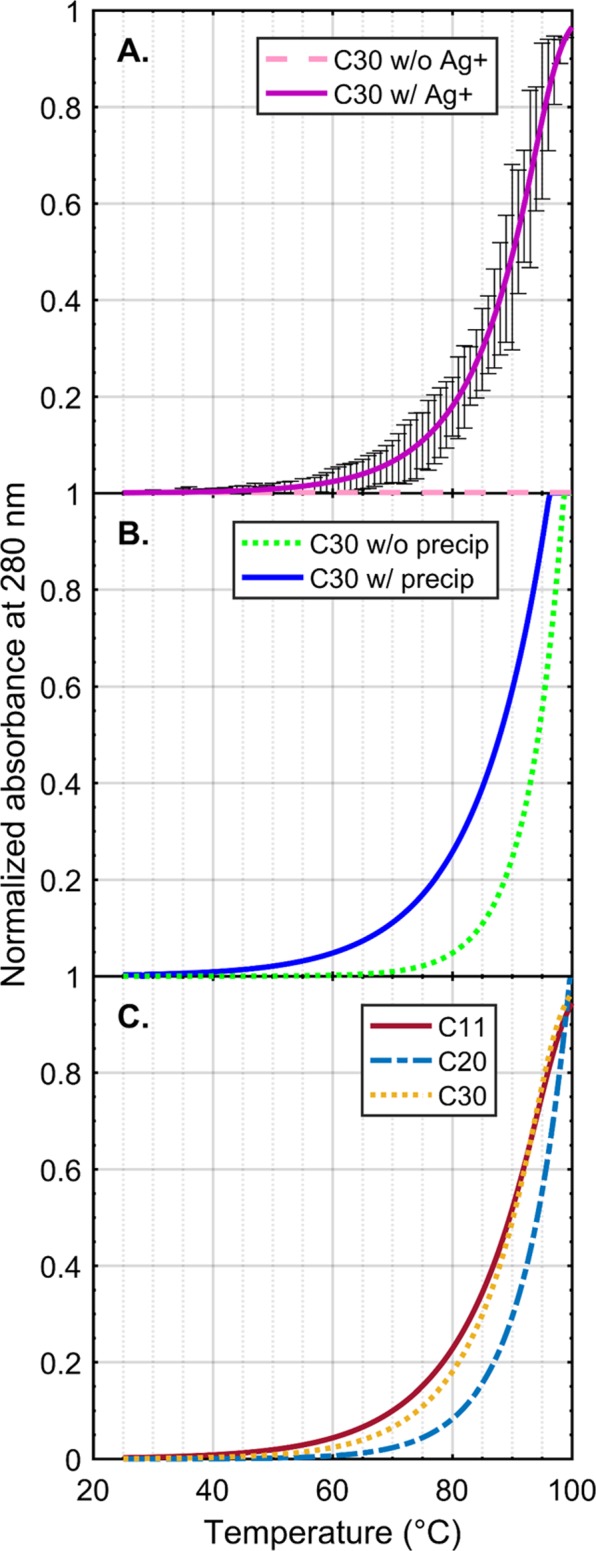
Melt curves of polycytosine oligomers. (**A**) The ion requirement for successful C30 melting is shown with standard deviation (n = 3). Without Ag^+^, no change in absorbance is shown over temperature, while the experimental condition shows full melting behavior characteristic of DNA duplexes. (**B**) Representative curves of C30 before and after precipitation of backbone-bound Ag^+^ as AgCl suggest a measurable difference in melting behavior, but that the primary contributor to duplex stability is the pyrimidine-coordinated cation chain. (**C**) Polycytosine oligomers of varying lengths show melting temperatures at or above 90 °C, indicating a powerful stabilizing effect mediated by the internal ion chains. The similarity between these melting temperatures likely reflects the maximum capacity of the instrument to assess thermal stability in water-based buffers.

We then investigated the stability of highly-mismatched polycytosine duplexes. We see from Fig. 5A that the C30 (30 nt polycytosine DNA) sequence, when annealed at a 10:1 Ag^+^:CC molar ratio, yields a melting profile with a melting temperature of 91 °C. The melting profile of the C30 sequence with Ag^+^ is far more articulated than the same C30 sequence without Ag^+^, as the latter lacks any ability to base pair without Ag^+^. Similarly, the melting profiles of C11 (11 nt polycytosine DNA) and C20 (20 nt polycytosine DNA) also produced melting temperatures of 90 °C and 93 °C, respectively (Fig. 5B). When compared with calculated melting temperatures of their canonical dC:dG duplex counterparts (62.5 °C, 83.3 °C, 90.7 °C, respectively)^75,76^, the Ag^+^-incorporated polycytosine chains are significantly more thermostable, especially with the shorter C11. It seems that the Ag^+^-intercalated polycytosine chains are resistant to temperatures approaching the boiling point of water. Further analysis was performed on the metalated C30 sample in the presence of excess NaCl, which precipitated uncoordinated Ag^+^ ions as AgCl (Fig. 5C). In agreement with the NMR experiments, exchanging the backbone-bound Ag^+^ cations with Na^+^ in this manner resulted in a small drop in melting temperature of <5 °C, but did not revert the melting profile to that of ssDNA. Ag^+^ thus may have a secondary role as a monovalent cation backbone stabilizer for duplex formation, similar to the well-documented effect of other cations, such as Na^+^ and Mg^2+^ ^77,78^.

**Figure 6:**
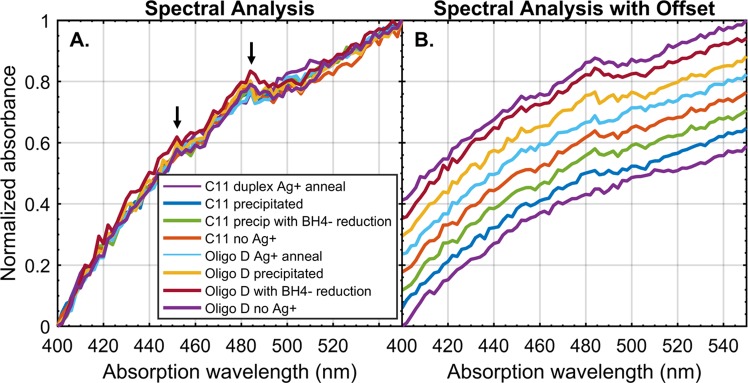
Normalized absorbance spectra of various annealed oligos from 400–550 nm. (**A**) Spectral overlay of C11 and Oligo D subjected to minus-Ag^+^ and positive-Ag^+^ conditions, precipitation of AgCl, and reduction by BH_4_^−^. Peaks are observed at 483 nm and 450 nm and appear to be universal across all conditions (black arrows). No significant perturbation is observed to suggest nanocluster formation. (**B**) The same data are offset visually for readability.

It has been shown previously that the reduction of Ag^+^ using single-stranded polycytosines as a template in the presence of the aggressive reducing agent borohydride (BH_4_^−^) causes a significant perturbation of the absorbance spectrum in the range of 450–500 nm due to nanocluster catalysis^79,80^. To assess whether complexed Ag^+^ ions along the backbone and coordinated by ring amines of cytosine-enriched sequences were reduced to DNA-bound silver nanoclusters, spectra of standard and cytosine-enriched oligonucleotides were collected at various stages of processing, both in the presence and absence of Ag^+^. Annealed duplexes were also subjected to forced reduction by BH_4_^−^ after precipitation to further assess the downstream behavior and solvent accessibility of pyrimidine-coordinated Ag^+^. Some DNA sequences featured 5′-amino modifiers in order to mimic STM experimental conditions described in conductivity studies^40,41^. A comparison between the C11 sequence and the Watson-Crick paired 28-bp DNA duplex Oligo D with and without AgNO_3_ showed no significant difference in the UV-visible absorption of the nucleotide solution (Fig. 6). Addition of BH_4_^−^ and precipitation of uncoordinated Ag^+^ ions did not affect the absorption spectra for either sample. In all tests, two peaks were observed in the critical range (450 nm and 483 nm), and this profile was conserved for all sequences, regardless of processing (Fig. S5). These results demonstrate that Ag^+^-intercalated polycytosine DNA duplexes exhibit high thermal stability without the drawback of undesirable nanocluster formation in common aqueous solutions.

### Synthesis of nucleic acid nanowires in synthetic biology

DNA polymerases and biased nucleotide pools can be used to synthesize nanowires in the presence of the mediating ion *in vitro*^81–83^. In the present study, we tested whether longer C-Ag^+^-C DNA duplexes can be constructed by traditional molecular biology methods in standard media. To this end, we carried out blunt-end ligation reactions on medium-length, double-stranded oligonucleotides. The 50-bp Oligo B duplex with six CC mismatches distributed throughout the sequence, using sections of Watson-Crick pairing to promote strand alignment and homogeneity between the products, thereby avoiding the chaining effect previously observed with the C20 and C30 sequences (Figs S1 and S2). In the absence of Ag^+^, a duplex product was formed but did not undergo double-stranded end-ligation, likely due to “puckering” at the mismatch points from unbonded nucleotides, which may cause bad strand alignment and inhibit ligase binding. When annealed in the presence of excess Ag^+^, 100, 150, 200, 250, and 300 bp dsDNA, bands were observed as a result of successful end-ligation (Fig. 2D). Ag^+^ intercalation between the mismatched cytosine pairs repaired the Oligo D duplex and enabled ligase recognition and activity that can extend the length of a DNA nanowire length more than six-fold.

**Figure 7:**
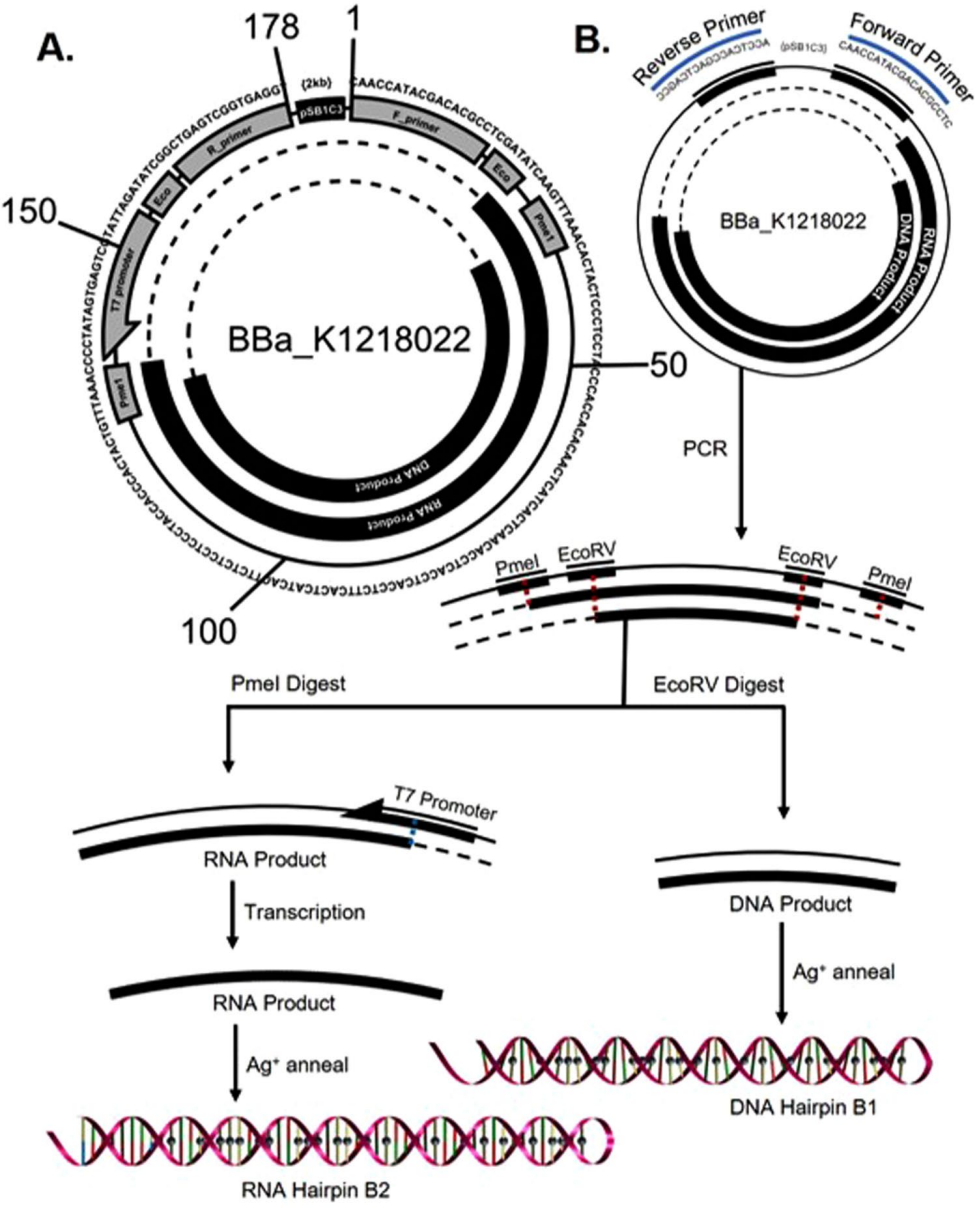
Part BBa_K1218022 serves as the template for DNA nanowire Hairpin B1 and RNA nanowire Hairpin B2. (**A**) Scheme is shown with sequence data, excluding the BioBrick prefix, suffix and plasmid backbone, collectively marked as “pSB1C3”, the expression plasmid backbone. Diagram includes the 178-bp functional region with forward and reverse primer binding sequences, EcoRV and Pme1 restriction sites, a T7 promoter and the two nanowire products. Concentric circles are employed to illustrate the different functions of gene part components. (**B**) To utilize this BioBrick, PCR amplification of the functional region should be performed using the identified promoters. The DNA nanowire product may be subsequently isolated by PmeI excision and annealed as described above. The template for RNA Hairpin B2 is excised by EcoRV and is designed to produce an RNA nanowire hairpin in a cell-free transcription system as described above.

To further expand the compatibility of core-functionalized DNA nanowires with living cells, we constructed two novel BioBricks for the synthesis of cytosine-mismatched, Ag^+^-binding nanowire templates in *E*. *coli* plasmids. BioBricks are a standard way to craft gene parts for general use through the open-source gene repository at the iGEM Registry. BioBricks are designed for modularity and compatibility with other gene parts, and are documented and curated by the BioBricks Foundation for ordering and use by registered labs^84^. PCR amplification and restriction digestion of part BBa_K1219026 produces both 32bp10CC Oligo A strands, which can be annealed into a duplex with a 10:1 Ag^+^:CC molar ratio. Similarly, PCR amplification of part BBa_K1218022 produces a DNA hairpin sequence with 24 cytosine mismatches (Fig. 7). Restriction digestion and T7 transcription of this template synthesizes a 54bp24CC RNA hairpin. These gene parts are the first templates for the synthesis of cytosine-enriched duplexes and hairpins in cell-free transcription systems with the use of PCR, and both parts and their respective implementations are documented in the iGEM registry for use by other groups.

## Discussion

We have presented a study of the structural, thermodynamic, and kinetic properties of Ag^+^-intercalated DNA duplexes for nanotechnology applications, and two methods for synthesizing DNA nanowires with standard synthetic biology techniques. In DNA strands with alternating cytosine mismatches and Watson-Crick base pairing, we have found that strands are annealed when Ag^+^ ions are equimolar with the number of cytosine mismatch sites. Ag^+^ ion incorporation between cytosine bases was visualized by 2D NMR, and the kinetics of incorporation was calculated from single-molecule fluorescence of hairpin formation to be 230-times slower than non-metalated hairpins. For polycytosine sequences, however, Ag^+^ ion concentration must be in at least 10-fold excess and the lack of Watson-Crick base pairing results in a loss of base pair “frame”, allowing polycytosine chains to develop randomly; these chains of interlocking strands can extend up to many times their individual strand length, in proportion to the environmental concentration of Ag^+^ ions. Metalated polycytosine chains have melting temperatures greater than their Watson-Crick duplex counterparts, even after accounting for counterion stabilization of the phosphate backbone. Notably, free Ag^+^ ions were not found to reduce to elemental Ag in the presence of DNA duplexes and form nanoclusters, even when the strong reducing agent BH_4_^−^ was added to the solutions.

The Ag^+^-intercalated DNA duplexes responded identically to canonical Watson-Crick base-paired duplexes in all of our structural probes. Silver, ethidium bromide, and toluidine blue staining protocols were used successfully to visualize Ag^+^-driven strand annealing by PAGE; NMR and melting curve profiles were entirely consistent with dsDNA; and Ag^+^-intercalated duplexes were recognized as substrates and successfully extended by T4 ligase. Ligation is especially noteworthy, indicating that multiple pyrimidine-ion pairs leave the overall helical structure relatively unperturbed, which agrees with reported structures of Ag^+^-intercalated DNA duplexes^57^. Predefined, medium-length Ag^+^-intercalated DNA duplexes can be synthesized by PCR amplification from standardized BioBrick plasmids and then elongated several-fold by enzymatic ligation. This result opens the door for downstream molecular biology reactions, and demonstrates that long nanowire synthesis can be achieved with definable length in this way through band excision or gel chromatography.

The primary difference between cytosine-mismatched and canonical DNA duplexes lies in the multimeric chaining behavior of polycytosine sequences. For these strands, high molecular weight streaks were discovered by PAGE at great Ag^+^ ion excess. This disparity suggests that ion overload causes long chain polymerization, perhaps due to local energy minima attained when all cytosines are ion-coordinated, even in a single-stranded state during high-temperature states in annealing. With lower ion availability, lower energy states may be more readily achieved by improving the annealing frame or strand alignment; with ion excess, these states may be unavailable or disfavored due to enthalpic penalties incurred from dislodging partial coordinating bonds or entropic penalties associated with ion rehydration.

Thermodynamically, Ag^+^ incorporation during annealing occurs spontaneously and is effectively irreversible under nondenaturing conditions. As indicated by the ^3^J_HH_ coupling of cytosine H5 and H6, the cytosine-cytosine mismatch easily incorporated a Ag^+^ ion after a relatively short reannealing procedure, but did not release the ion after being subjected to AgCl precipitation at room temperature and dialysis over several days at 6 °C. The difficulty of Ag^+^ ion extraction without denaturing the duplex suggests that the cytosine N3-Ag^+^ bond is shielded from the solvent in the interior of the duplex, as would be expected for this structure. In contrast, the chemical shifts of ribose H1′ and H2′/H2″ were much more labile; the Ag^+^-induced perturbations were mostly undone by the removal of excess Ag^+^ ions, so these effects were likely produced by electrostatic attraction between the Ag^+^ ions and negatively-charged phosphate backbones. The solvent accessibility of these bonds facilitates Ag^+^ ion release from the DNA backbone. This analysis is in agreement with the melting curve results, from which it is clear that the addition of Ag^+^ greatly increases the stability of polycytosine sequences, and that these oligomers are engaging in Ag^+^ ion uptake to form stable, duplex-like wires. The melting curves of Ag^+^-intercalated polycytosine chains also were mostly unaffected by the precipitation of excess Ag^+^, indicating that the vast majority of the stabilizing effect occurs as a result of nucleobase-ion coordination and not solvent-based electrostatic stabilization. These results suggest that Ag^+^-intercalated DNA nanowires could be assembled by simply incubating strands of DNA with soluble Ag salts and then washing off the unbound charges; the nanowire product would associate tightly with the Ag^+^ ions incorporated into the C-C mismatches, while the unbound ions would be removed. This is a highly desirable property for conducting nanowires to maintain an almost-linear conductive interior (Ag^+^ ion chain) while the exterior dielectric (solvent) remains resistive.

The corresponding kinetic analysis of Ag^+^ incorporation was performed by real-time single molecule fluorescence. The FRET states seen in these experiments represent distinct DNA conformations adopted in the presence and absence of Ag^+^ ions. By extrapolating the FRET efficiencies to approximate inter-fluorophore distances, we can predict likely DNA folds that would produce the observed FRET pattern (Fig. S6). Structures without Ag^+^-induced cytosine mismatches are limited to canonical base pairing, which is vulnerable to flexibility induced by large regions of single-stranded DNA. This flexibility manifests as broad FRET distributions as each molecule settles in a similar, yet distinct, local energy minimum. The more-populous Ag^+^-independent medium FRET state is likely produced by base pairing at the ends of the DNA strand, while the rare low FRET state lacks these pairs and, therefore, is only within FRET proximity transiently. The high FRET state that is only seen in the presence of Ag^+^ ions must reorder the canonical base pairs at the termini in order to allow C49 and C51 to pair with C8 and C10, respectively. The remaining cytosines on either side of the tetraloop are then in position to close the helix with the interspersed AT pairs. Accordingly, we propose that the Ag-induced high FRET state represents the intercalation of Ag^+^ ions into C-C mismatches at the measured folding rate of 0.065 s^−1^.

Practical considerations of using Ag^+^-intercalated DNA duplexes as biological nanowires demand that the unbound Ag^+^ ions not be involved in deleterious side reactions and that the wires be easily synthesized with sequence specificity and length control. The primary competing reaction for Ag^+^ ions in aqueous solutions is reduction, which can result in the polycytosine-catalyzed nucleation of silver nanoclusters^79,80^. The spectroscopic peaks in the 450–500 nm range that represent silver nanoclusters are not observed in this study. This is a departure from those studies on polycytosine-induced cluster nucleation which use exclusively isothermal reactions in saturating levels of reducing reagent. Our results indicate that the annealing conditions and processing experienced by duplexing DNA in the presence of Ag^+^ are not sufficient to cause nanocluster formation and that the ion competition and energetic stability of the metal-mediated, double-stranded structure can prevent reduction of internally coordinated ion chains by aqueous species. To further support this conclusion, electro-spray ionization mass spectroscopy (ESI-MS) has been used in the past to analyze the distribution of (Ag^+^)_N_-DNA products for homo-base oligomers, including C11^85^. Those results showed that the dominant product was, in fact, C_11_-(Ag^+^)_11_-C_11_, indicating that the silver ions participate primarily in a 1:1 base pair binding interaction. This supports the conclusion that the Ag^+^ ions are not reduced to nanoclusters along the chain, but rather assist in the duplex formation and contribute to base-pairing stability, ultimately suggesting that reduction contaminants are not responsible for the increase in molecular conductivity for precipitation-cleaned C11-amine duplexes reported previously^41^.

BioBricks, with standardized plasmids, can provide a convenient and inexpensive path toward the synthesis and scaled production of DNA nanowires. The BioBrick parts we have designed for this work require multiple processing steps, including enzymatic digestion, PCR, and Ag^+^ infusion. There are, however, no fundamental barriers preventing future designs from engineering around these steps. With the addition of mechanisms like Ag^+^ transporters, nanowire export proteins and tags, or co-transcriptional/reverse transcriptional assembly in cells^86,87^, future designs can be envisioned to produce useful constructs without direct intervention. Nonetheless, some obstacles still need to be overcome to ensure successful replication of more complex dC:Ag^+^:dC nanowires *in vivo*. Long template sequences with cytosine repeats will have complementary guanine-rich areas, which have been shown to assume a quadruplex conformation that may interfere with plasmid transcription and replication^88,89^.

Furthermore, any long nanowires or nanostructures assembled will be subject to a greater degree of base-pair promiscuity in the presence of Ag^+^, and will necessarily require careful sequence design, which may necessitate the development of new modeling tools. Some of these issues may be addressed by incorporating different metal base pairs together in a single sequence, including metalated dT:Hg^2+^:dT and dC:Ag^+^:dC pairs^54,90^, as well as other orthogonal pairs such as the imidazole-Ag^+^-imidazole or the Cu^2+^ complex bond^91,92^.

This issue also complicates the formation of nonlinear assemblies containing Ag^+^ base pairs, for which it will be necessary to minimize heterostructures while maximizing cytosine occupancy: this could also introduce additional challenges in WC pairing regions as a result of the emergent parity asymmetry between standard nucleotides. Standard origami scaffold M13mp18^20^ has 169,452 heterostructures greater than 3 bp in the absence of Ag^+^, the largest of which comprise five 12 bp dimers; conversely, when dC:dC bonds are facilitated by Ag^+^, the scaffold would have 295,755 heterostructures greater than 3 bp, including 21 size 12 bp dimers, two 13mers, one 14mer and two 16mers (analysis performed in Matlab, see Table S2). The calculated free energy of the largest homodimers are −20 kcal/mol and −31 kcal/mol, respectively, without adjustment for the free energy of Ag^+^ intercalation^75^. As such, it may be impractical to use native ssDNA plasmids without extensive mutagenesis to tailor the sequence for orthogonal chemical environments. Future studies may focus first on the assembly of short-oligonucleotide nanostructures^93,94^ using Ag^+^ pairing, rather than origami/staple interactions. Such studies will likely begin with extremely short oligomers with few heterostructures and work toward longer, more complex designs for plasmid integration.

The BioBricks presented here are an important first step toward microbial genes designed to build electrically-active nanowires from nucleic acids, and as such, demonstrate a preliminary approach to implementing a genetic system using orthogonal base chemistry in DNA with tools found naturally in and around *E*. *coli*. Bacterial chasses can provide highly reliable and homogenous production capabilities, and by harnessing their onboard manufacturing systems, significant progress can be made toward the directed integration of living biological systems into nanofabrication. Scaling of such a biologically-derived nanowire system would require only those materials involved in cell culture and DNA synthesis, as well as small amounts of ionic silver—thereby avoiding many of the resource- and reagent-associated barriers associated with silicon device technology. With this synthetic biological platform, we believe that the field of nanotechnology is capable of harnessing the favorable physicochemical properties of DNA to build effective, conductive, biological wires for use in self-assembling nanoelectronic architectures at reduced cost and increased scale.

## Supplementary information

Supplementary Information
