# Supplementary material for: Construction and characterization of metal ion-containing DNA nanowires for synthetic biology and nanotechnology
Source: Sci Rep. 2019 May 6;9:6942. doi: 10.1038/s41598-019-43316-1 (PMC6502794; doi:10.1038/s41598-019-43316-1)
Supplement: Supplementary file 1 — Supplementary Information [file 41598_2019_43316_MOESM1_ESM.pdf]

Construction and characterization of metal ion-containing DNA nanowires for synthetic biology and nanotechnology

Simon Vecchioni[1]*, Mark C. Capece[2,3]*, Emily Toomey[4]*, Nguyen Le[5], Austin Ray[6], Alissa Greenberg[7], Kosuke Fujishima[8], Jesica Urbina[9,10], Ivan G. Paulino-Lima[11], Vitor Pinheiro[12], Joseph Shih[13], Gary Wessel[14], Shalom J. Wind[15], Lynn Rothschild[10,14,†]

[1]Department of Biomedical Engineering, Columbia University, New York, NY 10027, USA.
[2]Department of Chemistry, Stanford University, Stanford, CA 94305, USA.
[3]Department of Structural Biology, Stanford University School of Medicine, Stanford, CA 94305, USA.
[4]Department of Electrical Engineering and Computer Science, Massachusetts Institute of Technology, Cambridge, MA 02139, USA.
[5]School of Engineering, Brown University, Providence, RI 02912, USA.
[6]Department of Chemical Engineering, Stanford University, Stanford, CA 94305, USA.
[7]Department of History, Stanford University, Stanford, CA 94305, USA.
[8]Earth-Life Science Institute, Tokyo Institute of Technology, Meguro-ku, Tokyo, 152-8550, Japan.
[9]Geology, Minerals, Energy, & Geophysics Science Center, U.S. Geological Survey, Menlo Park, CA 94025, USA.
[10]Planetary Science Branch, NASA Ames Research Center, Moffett Field, CA 94035, USA.
[11]Blue Marble Space Institute of Science, NASA Ames Research Center, Planetary Systems Branch, Moffett Field, CA, 94035-0001, USA.
[12]Institute of Structural and Molecular Biology, University College London, London, WC1E 6BT, UK.
[13]Department of Natural Sciences and Mathematics, University of Saint Mary, Leavenworth, KS 66048, USA.
[14]Department of Molecular Biology, Cell Biology, and Biochemistry, Brown University, Providence, RI 02912, USA.
[15]Department of Applied Physics and Applied Mathematics, Columbia University, New York, NY 10027, USA.

[†]Correspondence to Lynn Rothschild (lynn.j.rothschild@nasa.gov); alternate Simon Vecchioni (sav2123@columbia.edu)
*Contributed equally

Supporting Information: Figures for Publication

**Table S1.** Supplementary oligonucleotide sequences used in this study	
**Hairpin B1.** 50bp23CC hairpin	
Template	5'-ACACTACTCCCTCCTACCCACCACACAACTCATCACTCAACACCTCACCTCACCTCTTCACT CATCACTTCTCTCCTCCCTACCACCCACTACTGTTTAAA-3'
**Hairpin B2.** 54bp23CC RNA hairpin	
Template	5'-rCrArArGrUrUrUrArArArCrArCrUrArCrUrCrCrCrUrCrCrUrArCrCrCrArCrCrArCrArCrA rArCrUrCrArUrCrArCrUrCrArArCrArCrCrUrCrArCrCrUrCrArCrCrUrCrUrUrCrArCrUrCrArU rCrArCrUrUrCrUrCrUrCrCrUrCrCrCrUrArCrCrArCrCrCrArCrUrArCrUrGrUrUrUrArArArC rCrC-3'
**C40.** 40 nt polycytosine	
Template	5'-CCCCCCCCCCCCCCCCCCCCCCCCCCCCCCCCCCCCCCCC-3'
**C50.** 50 nt polycytosine	
Template	5'-CCCCCCCCCCCCCCCCCCCCCCCCCCCCCCCCCCCCCCCCCCCCCCCCCC-3'
**CG20.** 20 nt polycytosine and WC complement	
Template	5'-CCCCCCCCCCCCCCCCCCCC-3'
Complement	5'- GGGGGGGGGGGGGGGGGGGG-3'

Oligos in Table 1

C11

```
5'-CCCCCCCCCCC-3'
3'-CCCCCCCCCCC-5'

5'-CCCCCCCCCCC-3'
3'-CCCCCCCCCCC-5'
```

C20

```
5'-CCCCCCCCCCCCCCCCCCCC-3'
3'-CCCCCCCCCCCCCCCCCCCC-5'

5'-CCCCCCCCCCCCCCCCCCCC-3'
3'-…CCCCCCCCCC-5'3'CCCCCCCCCCCCCCCCCCCC-5'
```

C30

```
5'-CCCCCCCCCCCCCCCCCCCCCCCCCCCCCC-3'
3'-CCCCCCCCCCCCCCCCCCCCCCCCCCCCCC-5'

5'-CCCCCCCCCCCCCCCCCCCCCCCCCCCCCC-3'5'CCCCCCCCCC…-3'
3'-…CCCCCCCCCC-5'3'CCCCCCCCCCCCCCCCCCCCCCCCCCCCCC-5'
```

Oligo A

```
5'-TTATATTTACCACCTCCTCCACCTTTTAGATT-3'
3'-AATATAAATCCTCCACCACCTCCAAAATCTAA-5'
```

Oligo B

```
5'-TAAACCACTCATACCACAACAACTCTCTACTCCTACACATCATCCATCTC-3'
3'-ATTTGCTGACTATGGTGTTCTTGAGAGATCAGGATCTGTACTAGGTAGAG-5'
```

Oligo C

```
5'-TATTAAATAAAACTTTTATTTAATA-3'
3'-ATAATTTATTTTCAAAATAAATTAT-5'
```

Oligo D

```
5'-CATTAATGCTATGCAGAAAATCTTAG-3'
3'-GTAATTACGATACGTCTTTTAGAATC-5'
```

Hairpin A

```
5'-TTTGTGTCTCCTTCATCCCTCACTTTG T A
3'-ACACCAACTACCCACTCAAAC G T
```

Oligos in Table S1

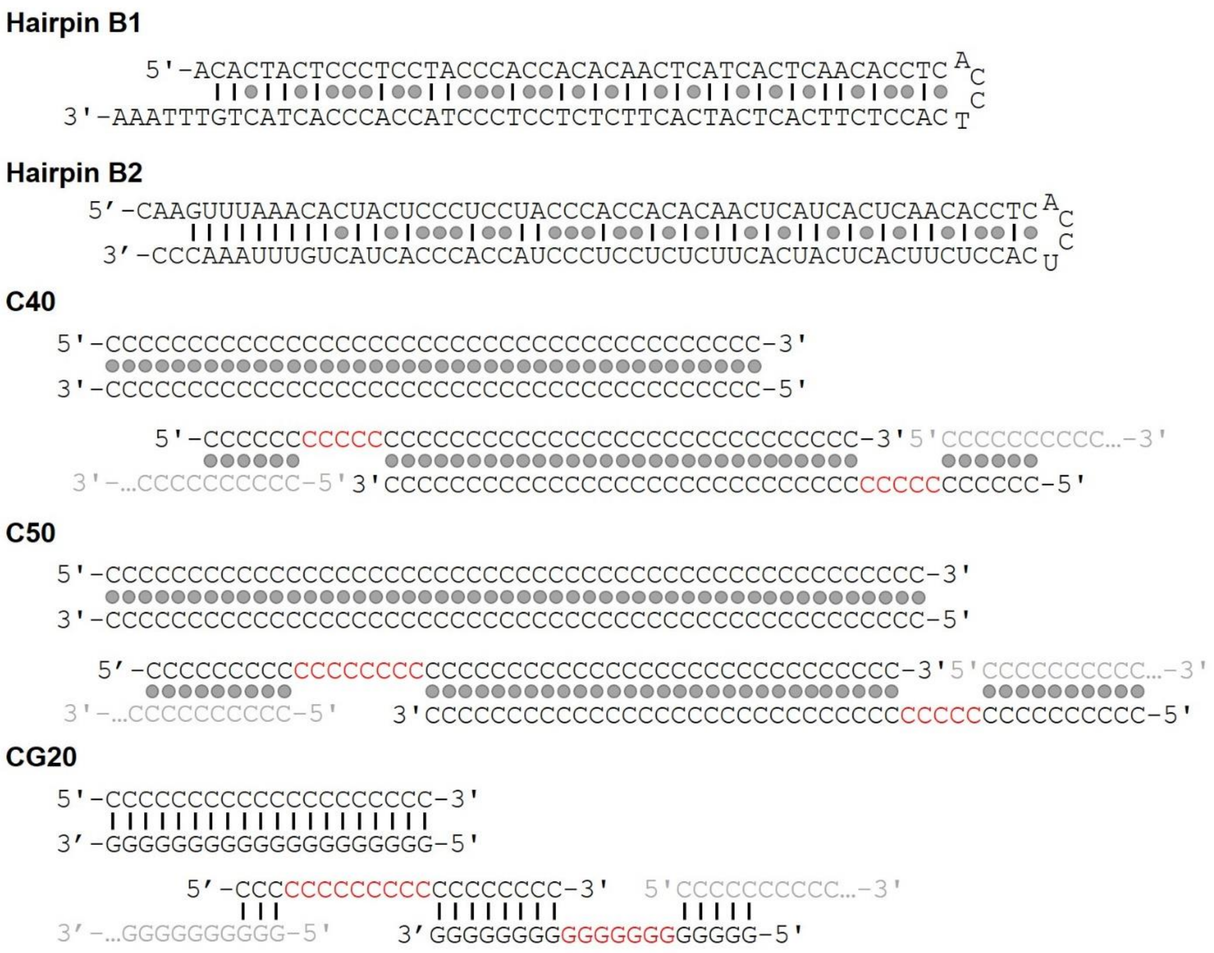

Figure S1. Predicted duplex and secondary structures for the oligos listed in Tables 1 and S1. Silver ions are shown as grey circles. In the case of the polycytosines, misaligned structures are shown as well, with unbound bases shown in red, and promiscuously-binding additional sequences shown in light gray. The Cy3 and Cy5 fluorophores are shown for Hairpin A. Linker chemistry is omitted.

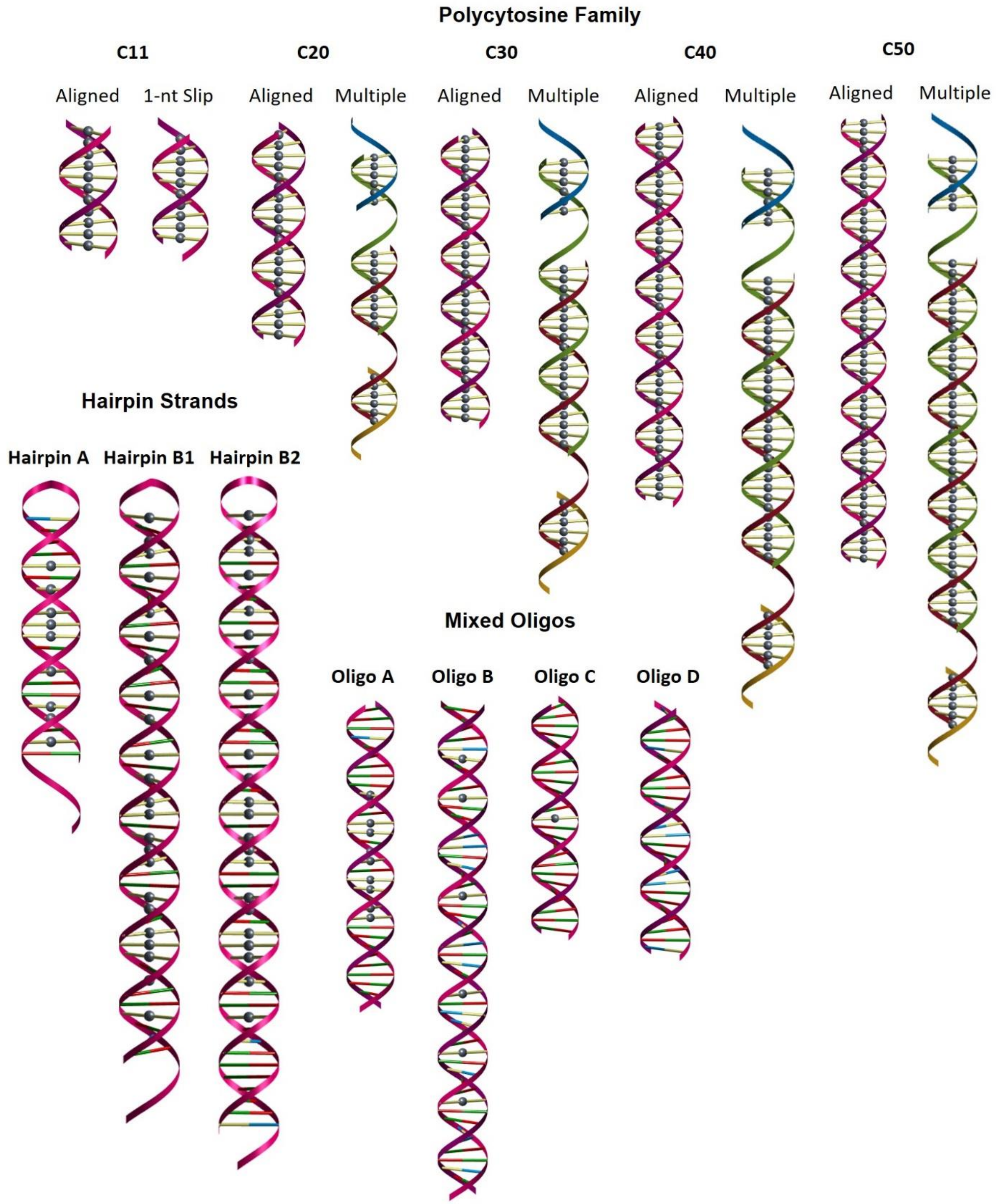

Figure S2. Rendering of the oligonucleotides in this manuscript (see Tables 1 and S1 and Fig. S1). Bases are color coded: guanine (blue), cytosine (yellow), adenine (green), thymine (red). Polycytosine structures

include the aligned annealing structure as well as a multiple-strand, non-unitary duplex. Models were implemented in Matlab (Mathworks, Natick, MA, USA).

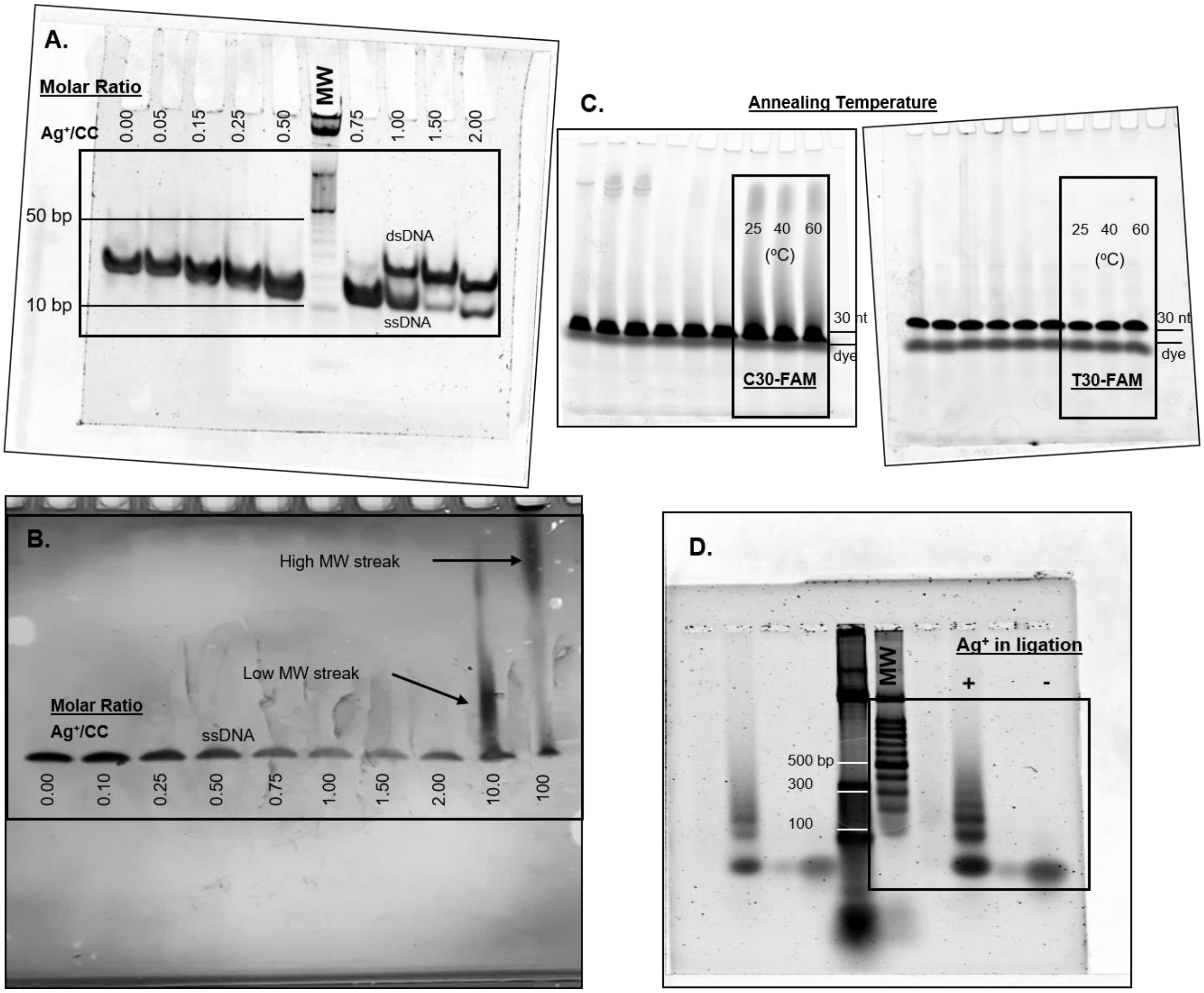

Figure S3. Same data as Figure 2 are shown uncropped. Cropped regions shown as inset dark box. Polyacrylamide gels demonstrate: A) Molar ratio of Oligo A duplex formation in the presence of Ag^+; B) Streaking behavior of C30 in the presence of high molar ratio Ag^+; C) Annealing of C30 but not T30 in the presence of Ag^+ at various temperatures; D) End-ligation of Oligo B after annealing with equimolar Ag^+. Images stained using SYBR Gold, silver staining kit, onboard fluorescein, and SYBR Gold, respectively, and are acquired using a Typhoon gel scanner.

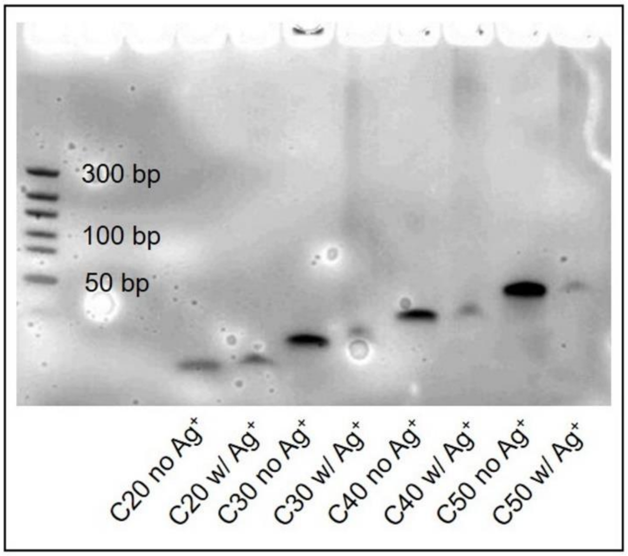

Figure S4. Polycytosines above 20 nt experience noticeable streaking into the 300+ bp range during annealing. PAGE gel visualized using toluidine blue after 30-minute post-stain after running.

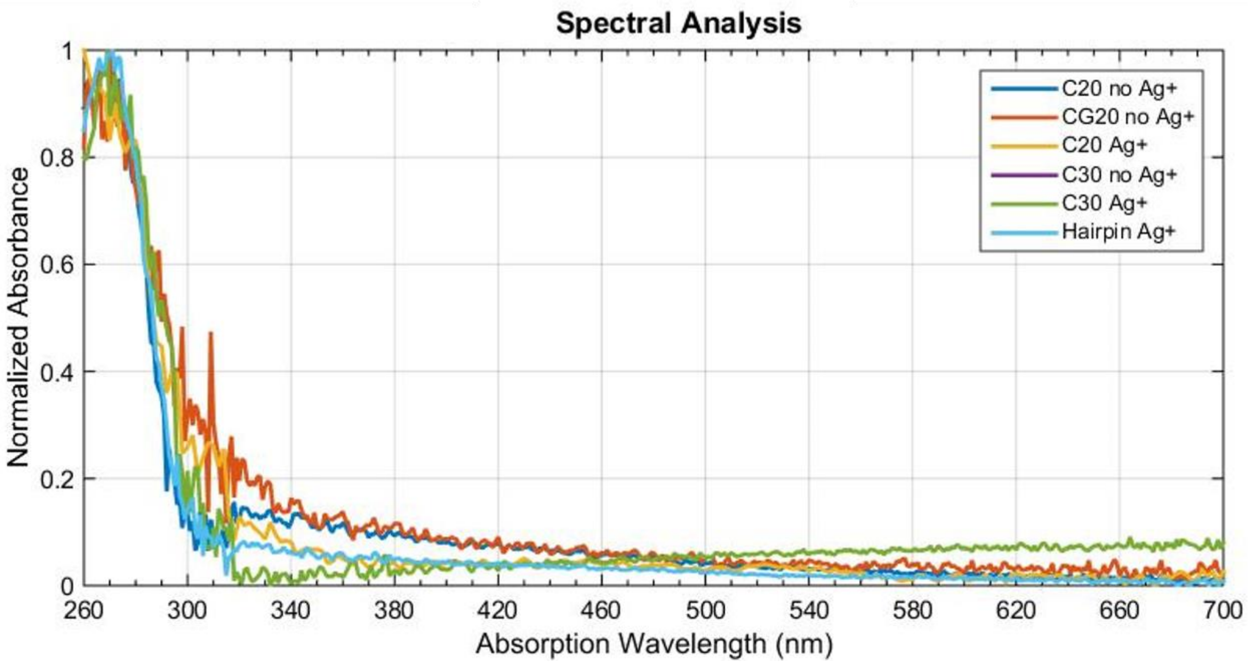

Figure S5. Additional normalized absorbance spectra of sequences C20, C30 and Hairpin A. The logical complement for C20, G20, is synthesized for canonical comparison (G30 was not commercially ordered due to tetraplex structures). No significant peaks are observed to indicate cluster formation across a variety of controls.

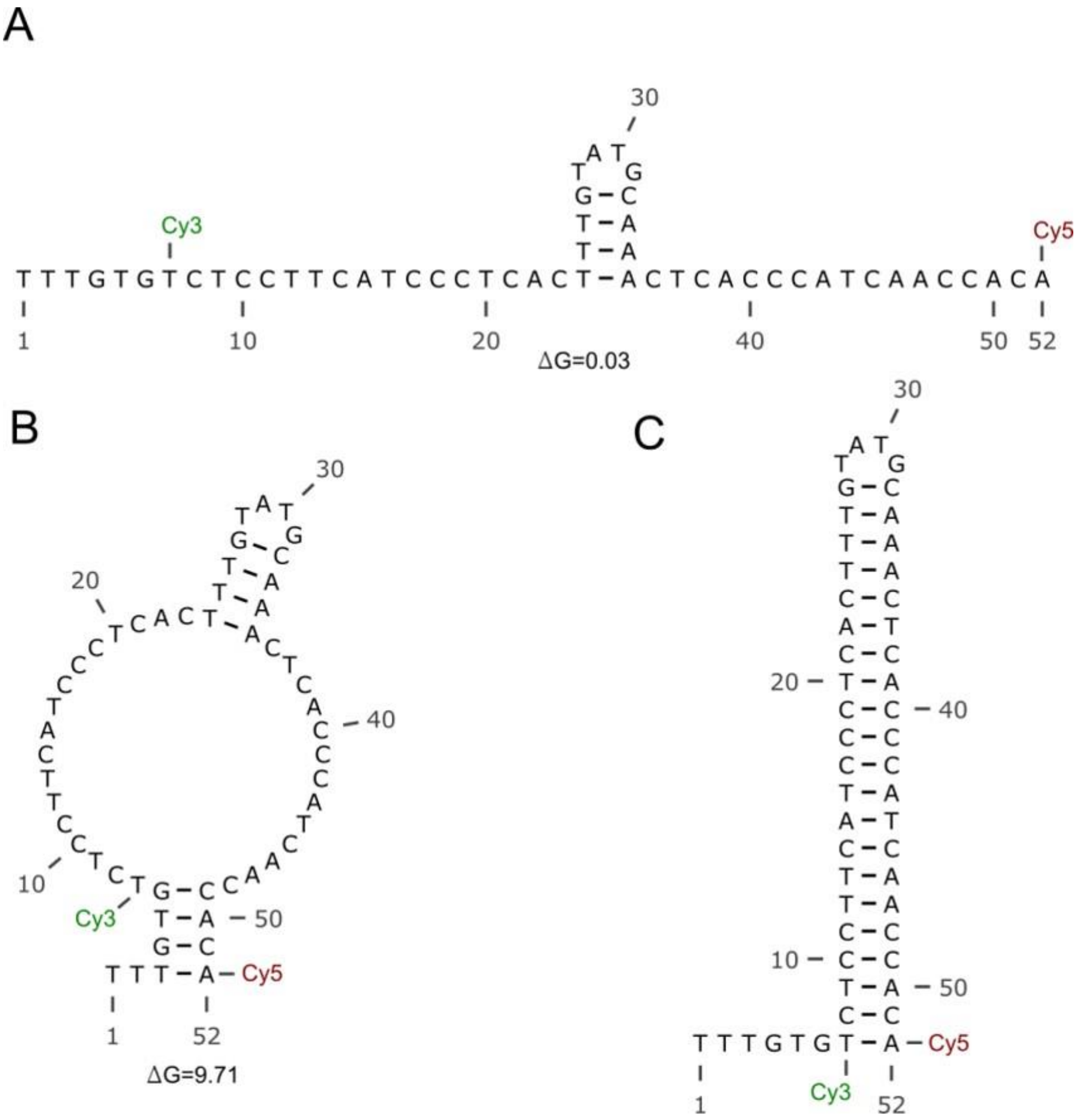

Figure S6. Secondary structures predicted to be adopted by the Hairpin A sequence from the FRET intensities measured by real-time single molecule fluorescence (Fig. 3). A) Primary structure of Hairpin A with the internal stem-loop and tetraloop emphasized. B) Most likely flexible secondary structure representing the low- and medium-FRET conformations possible without Ag^+ incorporation. C) Most likely rigid secondary structure representing the high-FRET conformation and requiring Ag^+ incorporation.

Table S2. Heterostructure analysis of M13mp18 ssDNA origami scaffold

Self-dimers in WC pairing environment													
**Size**	**4 bp**	**5 bp**	**6 bp**	**7 bp**	**8 bp**	**9 bp**	**10 bp**	**11 bp**	**12 bp**	**13 bp**	**14 bp**	**15 bp**	**16 bp**
**#**	125630	32200	8561	2270	581	152	35	18	5	0	0	0	0
Self-dimers in dC:Ag^{+}:dC pairing environment													
**Size**	**4 bp**	**5 bp**	**6 bp**	**7 bp**	**8 bp**	**9 bp**	**10 bp**	**11 bp**	**12 bp**	**13 bp**	**14 bp**	**15 bp**	**16 bp**
**#**	205527	62423	19489	5740	1775	567	140	68	21	2	1	0	2
